# Molecular Systematics of *Polygonum minus* Huds. Based on ITS Sequences

**DOI:** 10.3390/ijms12117626

**Published:** 2011-11-07

**Authors:** Hamidun Bunawan, Chee Yen Choong, Badrul Munir Md-Zain, Syarul Nataqain Baharum, Normah Mohd Noor

**Affiliations:** 1 Institute of Systems Biology, Universiti Kebangsaan Malaysia, Bangi 43600, Selangor, Malaysia; E-Mails: hamidunb@yahoo.com (H.B.); nataqain@ukm.my (S.N.B.); 2 School of Environmental and Natural Resource Sciences, Faculty of Science and Technology, Universiti Kebangsaan Malaysia, 43600 Bangi, Selangor, Malaysia; E-Mails: cychoong@ukm.my (C.Y.C.); abgbadd@ukm.my (B.M.M.-Z.); 3 School of Biosciences and Biotechnology, Faculty of Science and Technology, Universiti Kebangsaan Malaysia, 43600 Bangi, Selangor, Malaysia

**Keywords:** ITS, phylogenetics, plastid DNA, *Polygonum minus*, *trn*L-*trn*F

## Abstract

Plastid *trn*L-*trn*F and nuclear ribosomal ITS sequences were obtained from selected wild-type individuals of *Polygonum minus* Huds. in Peninsular Malaysia. The 380 bp *trn*L-*trn*F sequences of the *Polygonum minus* accessions were identical. Therefore, the *trn*L-*trn*F failed to distinguish between the *Polygonum minus* accessions. However, the divergence of ITS sequences (650 bp) among the *Polygonum minus* accessions was 1%, indicating that these accessions could be distinguished by the ITS sequences. A phylogenetic relationship based on the ITS sequences was inferred using neighbor-joining, maximum parsimony and Bayesian inference. All of the tree topologies indicated that *Polygonum minus* from Peninsular Malaysia is unique and different from the synonymous *Persicaria minor* (Huds.) Opiz and *Polygonum kawagoeanum* Makino.

## 1. Introduction

Current advances in plant molecular biology techniques and approaches provide a convenient and rapid evaluation of the differences in informational content, derived from DNA sequence data, of related individuals [1,2]. Polymerase chain reaction (PCR)-based techniques have been used comprehensively as plant molecular markers for significant phylogenetic presumption among relatively closely related species [3]. The impact of genetic data from chloroplast DNA (cpDNA) and nuclear ribosomal DNA (nrDNA) can be clearly seen in the fields of plant phylogenetics, systematics, population genetics and molecular biology [4,5].

*Polygonum minus* Huds., commonly known as kesum in Malaysia, is currently classified in the *Polygonum* section Persicaria. In 1967, Ridley [6] reported and described *P. minus* and another eight species of *Polygonum* from Malay Peninsula. No additional information on *P. minus* was available until Anjen *et al*. [7] and Freeman and Reveal [8] reported *Polygonum kawagoeanum* Makino and *Persicaria minor* (Huds.) Opiz, respectively, as being synonyms of *P. minus*. In 2010, we reported the chemical composition of essential oils in *P. minus* using two dimensional gas chromatography time of flight mass spectrometry (GC×GC-TOF MS) and found a significantly high level of aldehydes, especially decanal and dodecanal as the dominant aldehydes. Thus, *P. minus* appears to be more similar to *Polygonum odoratum* Lour., the Vietnamese coriander and *Persicaria hydropiper* L., known as the laksa plant in Singapore [9–11]. More recently, for the first time, we reported peltate glandular trichomes and conical brushlike clustered trichomes on both the adaxial and abaxial surfaces of *P. minus* and revealed potentially important distinctive features of the foliar micromorphology of the genus *Polygonum* [12]. Previously, Lerstern and Curtis [13] noted only capitate trichomes on the epidermis of *P. minus,* and they suggested trichome structure as a significant taxonomic feature in the genus *Polygonum*.

The taxonomic classification of the *Polygonum* genus has been debated since the work of Steward [14] and has presented a great taxonomic challenge [15]. Using cpDNA and nuclear ITS sequences, molecular phylogenetic studies within section Persicaria revealed that it is monophyletic and is most closely related to sections Tovara and Echinocaulon within the genus *Polygonum* [16]. To our knowledge, there has been no report on the genetic differentiation of *P. minus* individuals from Peninsular Malaysia. Therefore, in this study, we aim to highlight the potential impact of molecular genetic marker on selected *P. minus* accessions from Peninsular Malaysia based on the *trn*L-*trn*F and nuclear ribosomal ITS sequences in order to clarify the taxonomic status of *P. kawagoeanum* and *P. minor* as synonyms to *P. minus*.

## 2. Results and Discussion

PCR amplification of the *trn*L-*trn*F yielded a PCR product with a single-band of 380 bp in size, indicating the suitability of the universal PCR primers for *P. minus*. The *trn*L-*trn*F sequences among the four *P. minus* accessions were identical. This result implies that the *trn*L-*trn*F region from the plastid genome was not suitable to differentiate individuals of *P. minus.* The BLAST analysis against the GenBank database showed that the *trn*L-*trn*F sequences of *P. minus* had 100% similarity with sequences of *Persicaria pubescens* (EU197040.1), *Persicaria hydropiper* (EF653805.1) and *Persicaria longiseta* (EU109597.1). Therefore, the plastid DNA variation among members of the Persicaria section was low. However, plastid DNA variation has been successfully utilized in population genetics and biosystematic studies of other plants [17–19]. Okaura and Harada [20] reported the intraspecific variation in three non-coding regions of the plastid DNA in 21 Japanese beech (*Fagus crenata* Blume) populations and revealed the highly structured geographical distribution of cpDNA haplotypes.

**Figure 1:**
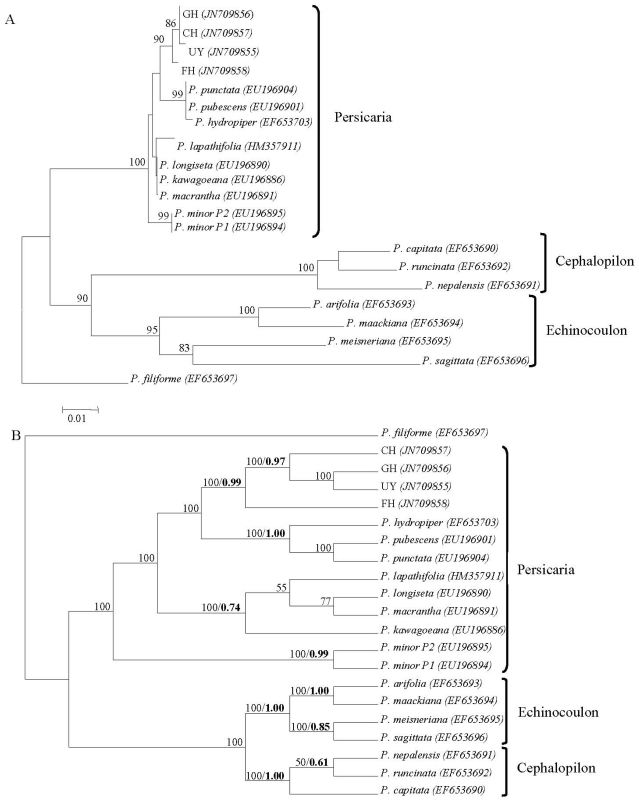
(**A**) The optimal Neighbor-Joining tree based on the kimura-2-parameter model. (**B**) One of the most parsimonious trees and Bayesian posterior probability tree based on ITS sequences. The values above the branches indicate bootstrap support (1000 replicates). Bayesian posterior probability values are indicated in bold.

The amplification of the ITS region produced a specific DNA band with a size of 650 bp. No sequence heterogeneity was detected among DNA clones from the same individual. The ITS sequences of the *P. minus* accessions were deposited into the GenBank database (JN709855-JN709858). The ITS sequence divergence among the four *P. minus* accessions was 1%, and it was more variable compared to the *trn*L-*trn*F sequences of *P. minus*. Generally, the plant nuclear genome evolves faster than the chloroplast genome [21]. The phylogenetic relationships among the *P. minus* accessions and selected *Persicaria* species based on the ITS sequences are shown in the neighbor-joining (NJ) tree [Figure 1(A)]. In the maximum parsimony (MP) analysis, 22.92% sites within the ITS region were found to be parsimony-informative characters. The MP analysis produced 18 most parsimonious trees with a tree length of 359 steps. One of the most parsimonious trees is shown in Figure 1B. The phylogenetic characteristics of ITS sequences from the MP analysis are summarized in Table 1. The topology in the Bayesian inference was similar to the NJ and MP trees (Figure 1).

The genetic distances between the *P. minus* (CH, GH, UY and FH), *P. kawagoeana*, *Persicaria minor* P1 and *P. minor* P2 were determined based on kimura 2-parameter model in PAUP. Genetic distance values are shown in Table 2 as percentages.

The tree topologies from the NJ, MP and Bayesian analyses were generally congruent with each other. The *Polygonum* taxa formed monophyletic lineages according to section Persicaria (*P. punctata*, *P. pubescens*, *P. hydropiper*, *P. lapathifolia*, *P. longiseta*, *P. kawagoeana*, *P. macrantha* and *P. minor*), section Cephalopilon (*P. capitata*, *P. runcinata* and *P. nepalensis*) and section Echinocoulon (*P. arifolia*, *P. maackiana*, *P. meisneriana* and *P. sagittata*). All four of the *P. minus* accessions [Cameron Highlands (CH), Genting Highland (GH), Fraser’s Hill (FH) and Ulu Yam (UY)] from Peninsular Malaysia formed a clade that was strongly supported by bootstrap value and posterior probabilities (Figure 1). The MP tree topology showed that the Peninsular Malaysian *P. minus* clade formed a close relationship to a clade containing *P. pubescens, P. hydropiper* and *P. punctata* (Figure 1).

Although *Persicaria minor* and *Polygonum kawagoeanum* are treated as synonyms of *Polygonum minus* [7,8], these three taxa did not cluster together in any of the constructed trees (Figure 1). The three species were found to have great genetic distances (Table 2). *Polygonum minus* had the highest genetic distance from *P. kawagoena* and *P. minor*. Therefore, *P. minus* of Peninsular Malaysia is exclusive and unique, and it might be distinct from the synonymous *P. minor* and *P. kawagoeanum*.

## 3. Experimental Section

### 3.1. Plant Materials

Fresh leaves of wild-type *Polygonum minus* were collected in June 2009 from Genting Highland, Cameron Highlands and Fraser’s Hill in Pahang and Ulu Yam in Selangor. The morphology and anatomy of the wild accessions were examined and confirmed as *P. minus* as described in our previous study of the plant [12]. Accessions of Genting Highland (GH) and Cameron Highlands (CH) have large leaves, while accessions of Fraser’s Hill (FH) and Ulu Yam (UY) have small leaves. Voucher specimens were deposited in the Herbarium Universiti Kebangsaan Malaysia (UKMB), Bangi, Malaysia.

### 3.2. Genomic DNA Isolation

*P. minus* genomic DNA was isolated from fresh leaf tissue according to the methods of Doyle and Doyle [22] and Cullings [23]. The quantification of genomic DNA was achieved using UV–Visible spectroscopy (Varian, Australia) by measuring the absorbance at A_260_, A_280_ and A_320_ nm. DNA purity was determined by the A_260_/A_280_ absorbance ratio and tested by running the genomic DNA samples on a 1% agarose gel stained with 0.25 μg/mL ethidium bromide. The gel was visualized and photographed under UV light using a Fujifilm LAS-3000 imager.

### 3.3. PCR Amplification

PCR amplification was performed in a 50 μL volume containing 0.2 mM of each dNTP, 1× PCR reaction buffer (1.5 mM MgCl_2_), 1.0 μM of each primer, 1.25 U *Taq* DNA polymerase (Promega) and 0.5 μg/50 μL template DNA. The *trn*L-*trn*F region was amplified with primers 5′-GGT TCA AGT CCC TCT ATC CC-3′ and 5′-ATT TGA ACT GGT GAC ACG AG-3′ [24]. The primers ITS-leu 5′-GTC CAC TGA ACC TTA TCA TTT AG-3′ [25] and ITS4 5′-TCC TTC CGC TTA TTG ATA TGC-3′ [26] were used to amplify the ITS region. Amplification of the *trn*L-*trn*F and ITS regions was carried out using the following thermal cycle profile: primary denaturation for 5 min at 94 °C, followed by 30 cycles of 1 min at 95 °C, 1 min at 55 °C and 1 min at 72 °C, and a final extension of 5 min at 72 °C.

### 3.4. PCR Product Purification, Cloning and Sequencing

The PCR products of *trn*L-*trn*F and ITS were checked on a 1% agarose gel. The PCR products were then purified using the Gel Extraction Kit (Qiagen, Valencia, CA, USA). For ITS, the PCR products were further cloned by using the PGEM-T Easy Vector System (Promega, Madison, WI, USA). Minipreps of plasmid DNA were made with the Qiagen Miniprep Kit, followed by restriction analysis to identify the clones for sequencing. Ten positive clones were selected for sequencing using the SP6 Promoter Primer and T7 Promoter Primer to check for sequence heterogeneity. DNA cycle sequencing was performed using the ABI Prism Dye Terminator Cycle Sequencing Ready Reaction kit and analyzed with the ABI PRISM 3100 Genetic Analyzer (Perkin-Elmer, Waltham, MA, USA).

### 3.5. Data Analyses

The ITS sequences for the four *P. minus* accessions of Peninsular Malaysia (GH, CH, FH and UY) and 16 other *Polygonum* species acquired from the GenBank database [*Persicaria pubescens* (EU196901), *Persicaria hydropiper* (EF653703), *Persicaria punctata* (EU196904), *Persicaria lapathifolia* (HM357911), *Persicaria longiseta* (EU196890), *Persicaria macrantha* (EU196891), *Persicaria kawagoeana* (EU196886.1), *Persicaria minor* P1 (EU196894), *Persicaria minor* P2 (EU196895), *Persicaria capitata* (EF653690), *Persicaria runcinata* (EF653692), *Persicaria nepalensis* (EF653691), *Persicaria arifolia* (EF653693), *Persicaria maackiana* (EF653694), *Persicaria meisneriana* (EF653695) and *Persicaria sagittata* (EF653696)] were used in phylogenetic analyses. The obtained nucleotide sequences were aligned by the ClustalX Multiple Sequence Alignment programme [27] using the default settings. The maximum parsimonious (MP) and neighbor-joining (NJ) trees were then generated using PAUP* 4.0b10 [28], and the Bayesian inference was done by MrBayes 3.1 [29]. The genetic distances were determined based on the kimura 2-parameter model using the default settings in PAUP* 4.0b10 and were used in generating the NJ tree. The MP analysis was conducted using a heuristic search with the TBR branch-swapping algorithm, and gaps were treated as missing data. Bootstrap analyses with 1000 replicates were conducted to obtain confidence in the NJ and MP trees. Consistency and retention indices (CI and RI, respectively) of the MP tree were generated using PAUP* 4.0b10. Modeltest 3.7 [30] was used to select the substitution model that best fit the data using the AIC criterion. The best model was subsequently used for Bayesian analysis in MrBayes 3.1 (TVM + G, with a proportion of invariable sites of 0 and a gamma distribution shape parameter of 0.1092). Bayesian inference was made by running two simultaneous metropolis-coupled Monte-Carlo Markov chains for 2,000,000 generations with an average standard deviation of split frequencies of 0.003639. The consensus topology tree of 9001 was produced by omitting the first 1000 trees of 10,000 (burning) with a tree that was sampled for every 100 generations. *Polygonum filiforme* (EF653697) was used as an outgroup taxon in the phylogenetic analyses.

## 4. Conclusions

In *P. minus*, the ITS region from the nuclear genome was more variable than the *trn*L-*trn*F region from the plastid genome. The ITS sequences were informative enough to infer the phylogenetic relationships of *P. minus* and the related *Polygonum* taxa. The topology of the NJ, MP and Bayesian consensus trees showed that *P. minus* from Peninsular Malaysia did not form a cluster with *P. kawagoeana* and *P. minor*, which were previously reported to be synonyms to *P. minus*. These findings suggest that further investigation is needed to clarify the taxonomic status of these three taxa.

## Figures and Tables

**Table 1:** Phylogenetic characteristics of ITS sequences for maximum parsimony (MP) analysis.

Characteristic	Value
Tree length	359
Consistency index (CI)	0.7744
Homoplasy index (HI)	0.2256
CI excluding uninformative characters	0.7254
HI excluding uninformative characters	0.2746
Retention index (RI)	0.8247
Rescaled consistency index (RC)	0.6386

**Table 2:** Genetic distance percentages between *Polygonum minus*, *P. kawagoeana*, *P. minor* P1 and *P. minor* P2.

Species	*Polygonum minus*	*P. kawagoeana*	*P. minor P1*	*P. minor P2*
*Polygonum minus*	-	0.95	1.65	16.5
*P. kawagoeana*	0.95	-	0.9	0.9
*P. minor P1*	1.65	0.90	-	0.0
*P. minor P2*	1.65	0.90	0.0	-
